# Supplementary material for: Electrochemical and Mechanistic Study of Reactivities of α-, β-, γ-, and δ-Tocopherol toward Electrogenerated Superoxide in N,N-Dimethylformamide through Proton-Coupled Electron Transfer
Source: Antioxidants (Basel). 2021 Dec 22;11(1):9. doi: 10.3390/antiox11010009 (PMC8773314; doi:10.3390/antiox11010009)
Supplement: Supplementary file 1 [file antioxidants-11-00009-s001.zip › antioxidants-1515673-supplementary.pdf]

Supplementary data for

Electrochemical and Mechanistic Study of Reactivities of α-, β-, γ-, and δ-Tocopherol Toward Superoxide in *N*,*N*-Dimethylformamide through Proton-Coupled Electron Transfer

Tatsushi Nakayama [1]*, Ryo Honda [2], Kazuo Kuwata [2], Shigeyuki Usui [1], and Bunji Uno [3]

[1] Department of Pharmacy, Gifu Pharmaceutical University, 1-25-4 Daigaku-nishi, Gifu 501-1196, Japan

[2] United Graduate School of Drug Discovery and Medical Information Sciences, Gifu University, 1-1 Yanagido, Gifu 501-1193, Japan

[3] Faculty of Pharmacy, Gifu University of Medical Science, 4-3-3 Nijigaoka, Kani, Gifu 509-0923, Japan

* Correspondence: tnakayama@gifu-pu.ac.jp

Table of contents

Pages S2	Figure S1; scheme of the *in-situ* electrolytic ESR spectral system
	Table S1; comparisons of the Δ*G*°s for two mechanisms
Pages S3	Figure S2; change in HOMO−LUMO energies
Pages S4	Figure S3; energy profiles along ET–PT via TS between TOH and $HO_2^{\bullet}$
Pages S5	Figure S4: Plausible PCET pathways between $O_2^{\bullet -}$ and α-TOH
	Figure S5: Plausible PCET pathways between $O_2^{\bullet -}$ and γ- TOH.

Pages S6-S13 Table S2-S5: Calculated geometries of the complexes formed between TOH and $O_2^{\bullet-}$ with DFT-B3LYP/PCM/6-311+G(d,p)

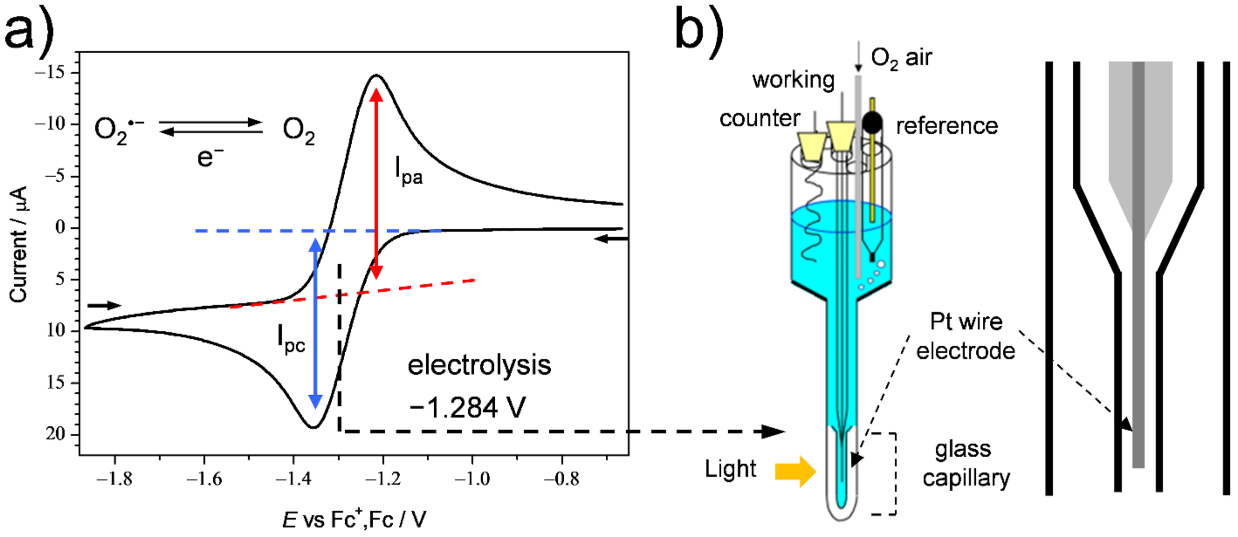

Figure S1. *In situ* electrolytic ESR spectral system. (a) Cyclic voltammograms of $O_2/O_2^{\bullet-}$ for potential determination. (b) *In* situ ESR system, composed of an electrochemical ESR cell with a glass small tip, air tube for O_2 bubbling, and three electrode system using a 0.5-mm-diameter straight Pt wire sealed in a glass capillary as working electrode.

Table S1. Free energy changes ($\Delta G°$/kJ mol^{-1}, 298.15 K) of ET between (a) TO^- and $HO_2^{\bullet}$ (along intermolecular ET–PT), and between (b) TOH and $HO_2^{\bullet}$ (along PCET), in DMF.

	α-TO⁻/α-TOH	β-TO⁻/β-TOH	γ-TO⁻/γ-TOH	δ-TO⁻/δ-TOH
TO^- and $HO_2^{\bullet}$	−50.3	−40.1	−38.6	−28.4
TOH and $HO_2^{\bullet}$	100.7	103.3	111.4	117.1

[1] $\Delta G°$s were calculated using DFT at the (U)B3LYP/PCM/6-311+G(d,p) level. [2] Electron transfer (ET), proton transfer (PT), proton-coupled electron transfer (PCET).

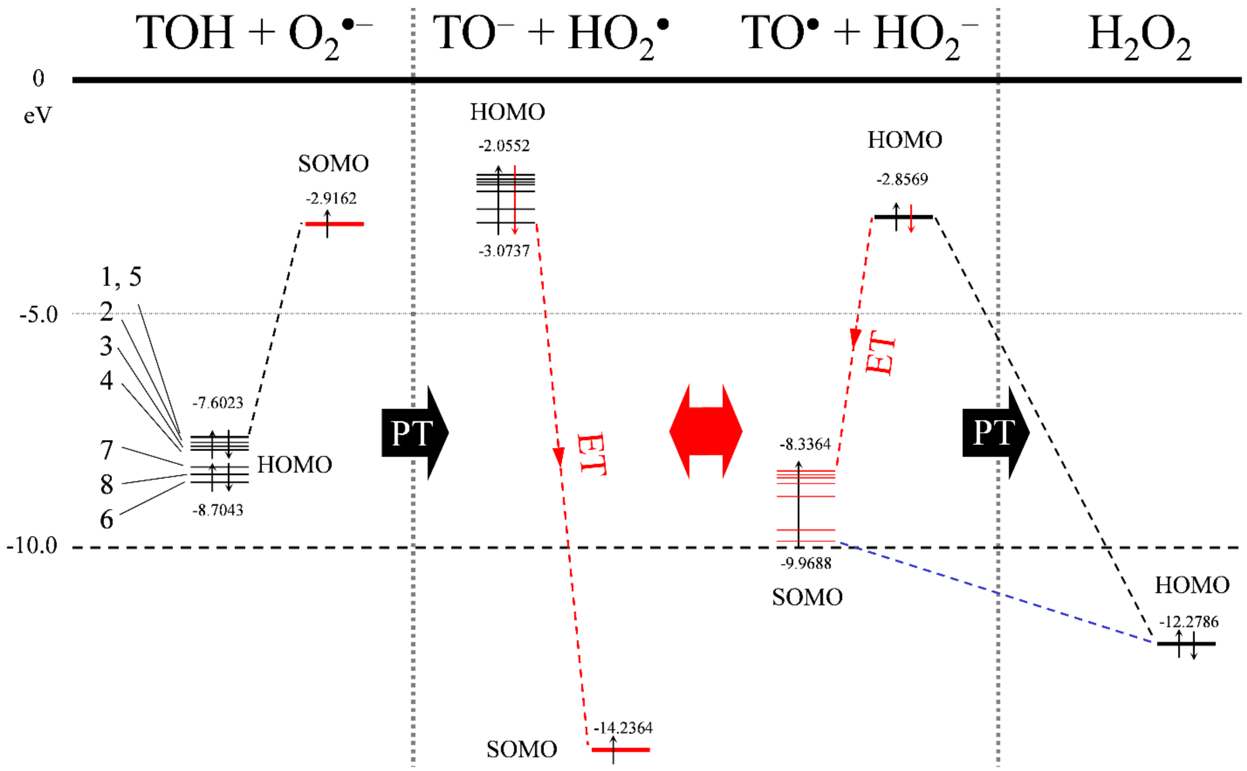

Figure S2. Change in highest occupied molecular orbital-lowest unoccupied molecular orbital (HOMO—LUMO) energies (E_h/a.u.) along the $O_2^{\bullet-}$ elimination reaction (the first proton transfer (PT), electron transfer (ET), and the second PT) by (a–d) α-, β-, γ-, and δ-TOH, (e) 2,2,5,7,8-pentamethyl-6-chromanol, (f) homogentisic acid γ-lactone, (g) 2,3-dihydro-2,2-dimethyl-7-hydroxybenzofuran, and (h) trans-*para*-coumaric acid, calculated with the HF/6-311+G(d,p) method.

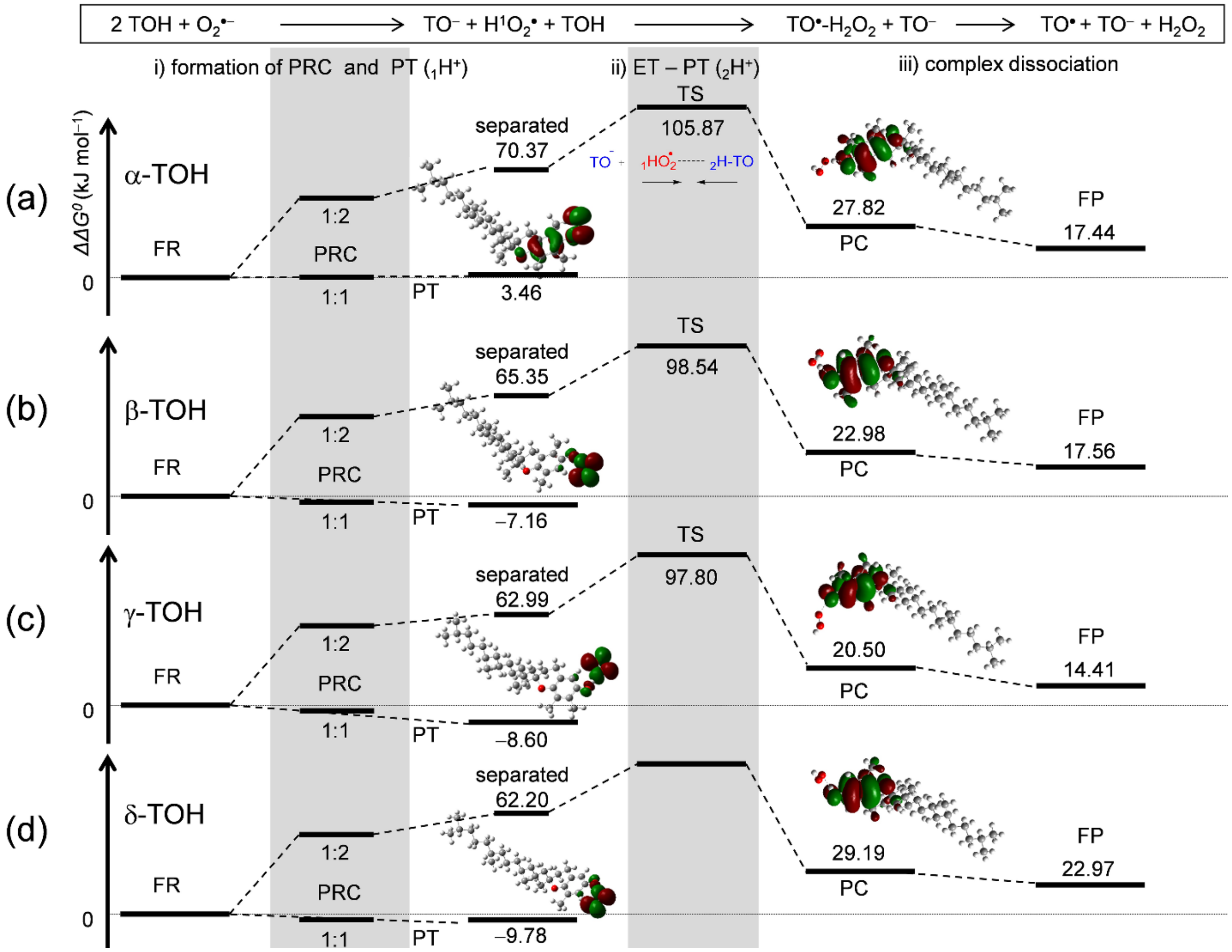

Figure S3. Energy profiles along PCET involving two PTs and one ET between two molecule of TOH (α-, β-, γ-, and δ-TOH) and $O_2^{\bullet-}$ in DMF, calculated using DFT-(U)B3LYP/PCM/6-311+G(d,p) method. Activation energies (kJ mol^{-1}) of transition states (TS) were obtained for the 1:1 ET-PT pathway between TOH (α-, β-, and γ-TOH) and $HO_2^{\bullet}$.

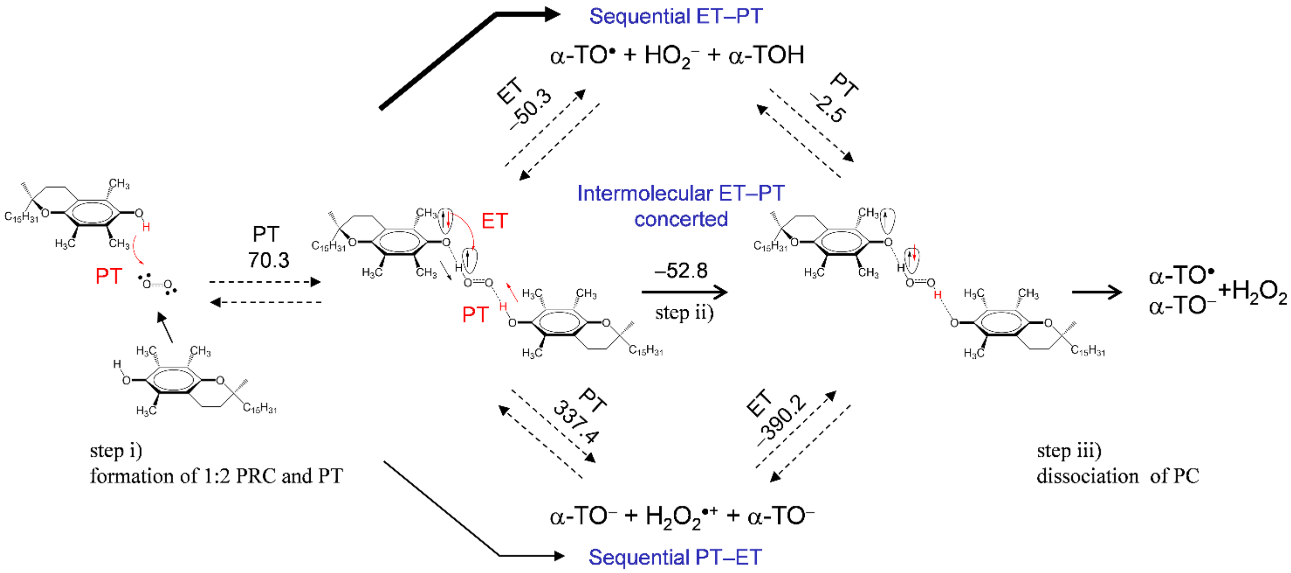

Figure S4. Plausible mechanism and the Δ*G*°s (kJ mol^{-1}, 298.15 K) for the PCET pathways between $O_2^{\bullet-}$ and α-TOH involving two PTs and one ET in DMF. The Δ*G*°s were calculated using DFT-(U)B3LYP/PCM/6-311+G(d,p) method.

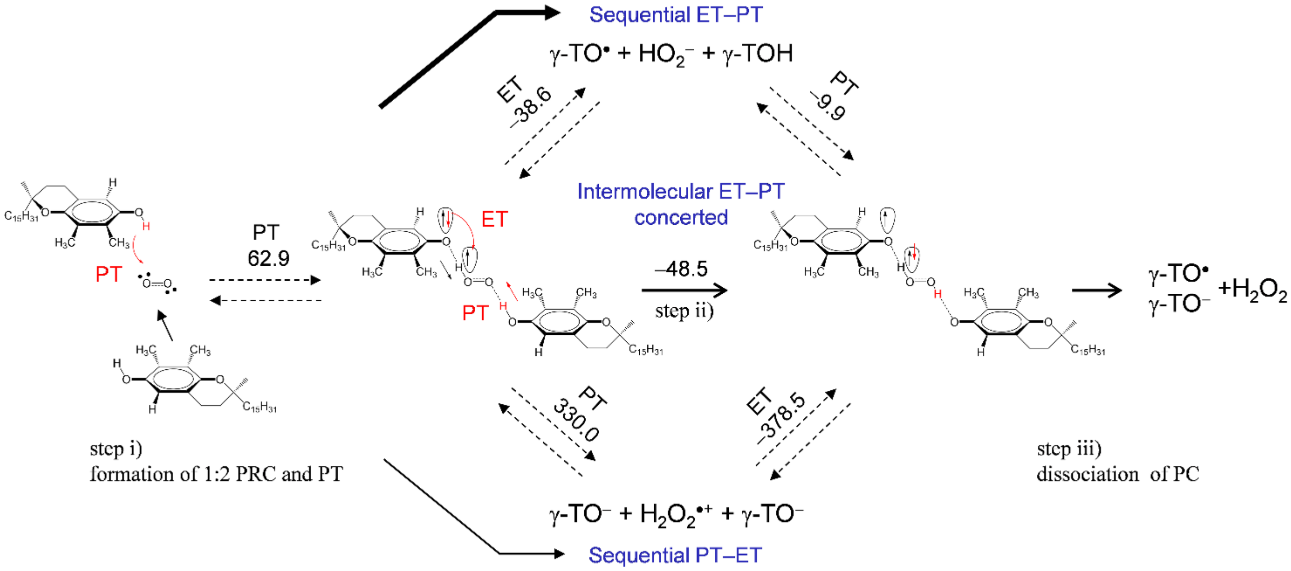

Figure S5 Plausible mechanism and the Δ*G*°s (kJ mol^{-1}, 298.15 K) for the PCET pathways between $O_2^{\bullet-}$ and γ-TOH involving two PTs and one ET in DMF. The Δ*G*°s were calculated using DFT-(U)B3LYP/PCM/6-311+G(d,p) method.

Table S2. Calculated geometries of the complexes formed between (a) α-TOH and $O_2^{\bullet-}$, (b) α-TOH and $HO_2^{\bullet}$ in DMF, with DFT-(U)B3LYP/PCM/6-311+G(d,p).

	(a) α-TOH and $O_2^{\bullet-}$				(b) α-TOH and $HO_2^{\bullet}$			
Center Number	Atomic Number	Coordinates (Angstroms)			Atomic Number	Coordinates (Angstroms)		
		X	Y	Z		X	Y	Z
1	6	5.17795	-0.0119	1.34123	6	-5.0866	-0.1098	-1.35
2	6	4.89373	-0.9748	0.35804	6	-4.6677	-1.0633	-0.3621
3	6	5.32702	-0.8179	-0.9642	6	-5.094	-1.0121	0.98897
4	6	6.02599	0.34804	-1.3261	6	-5.9012	0.03364	1.39451
5	6	6.31705	1.31594	-0.3504	6	-6.3266	1.03864	0.43294
6	6	5.91992	1.12752	0.98927	6	-5.8955	0.93479	-0.9592
7	6	5.02668	-1.8877	-1.9931	6	-4.6682	-2.1031	1.94516
8	1	4.27648	-1.5185	-2.7035	1	-3.9051	-1.7176	2.63074
9	1	5.91956	-2.0903	-2.5921	1	-5.5148	-2.3895	2.57385
10	6	6.46693	0.55579	-2.7562	6	-6.3725	0.15468	2.81674
11	1	7.26094	-0.1452	-3.0402	1	-7.1045	-0.6251	3.05761
12	1	5.64171	0.3979	-3.4575	1	-5.5443	0.04261	3.52193
13	1	6.84898	1.56538	-2.8935	1	-6.8464	1.12077	2.97794
14	6	4.70754	-0.2185	2.76305	6	-4.5889	-0.3277	-2.7581
15	1	3.92839	-0.9774	2.80729	1	-4.8335	-1.3369	-3.0987
16	1	5.52594	-0.5484	3.41486	1	-5.017	0.37964	-3.4629
17	1	4.31391	0.70696	3.1906	1	-3.4997	-0.2387	-2.8032
18	6	6.30453	2.13599	2.04789	6	-6.3861	2.01883	-1.8806
19	1	6.63448	1.63961	2.96409	1	-5.9728	1.94245	-2.8827
20	1	7.11259	2.77882	1.70058	1	-7.4774	1.99258	-1.9623
21	1	5.4653	2.78647	2.31983	1	-6.1254	3.00349	-1.4832
22	6	4.54859	-3.1904	-1.3476	6	-4.1518	-3.3349	1.20045
23	1	4.09437	-3.8461	-2.0958	1	-3.6615	-4.0243	1.8918
24	1	5.40532	-3.7273	-0.927	1	-4.9942	-3.8723	0.75373
25	6	3.55316	-2.9388	-0.2068	6	-3.1747	-2.9677	0.08083
26	8	4.20698	-2.0972	0.78519	8	-3.8527	-2.0212	-0.8297
27	6	3.25079	-4.2406	0.53309	6	-2.8696	-4.177	-0.7971
28	1	4.18105	-4.7299	0.83152	1	-3.7967	-4.6499	-1.1279
29	1	2.65886	-4.0561	1.43108	1	-2.2943	-3.8914	-1.6784
30	1	2.69301	-4.9228	-0.1137	1	-2.2908	-4.9094	-0.2293
31	6	2.26979	-2.2468	-0.7163	6	-1.9016	-2.2856	0.62058
32	1	2.55021	-1.3108	-1.2095	1	-2.1943	-1.4003	1.1939
33	6	1.20385	-1.9368	0.34274	6	-0.8576	-1.8696	-0.4241
34	1	0.81937	-2.8677	0.77434	1	-0.4617	-2.7552	-0.9326
35	1	1.66891	-1.3807	1.16137	1	-1.3415	-1.2573	-1.1899
36	8	7.04163	2.4191	-0.7245	8	-7.0748	1.98978	0.8065
37	1	6.61288	3.29266	-0.4234	1	-7.9013	3.33861	0.18293
38	1	1.83796	-2.8857	-1.4961	1	-1.4472	-2.9783	1.33835
39	6	0.02765	-1.1419	-0.2413	6	0.30935	-1.1015	0.21184
40	1	-0.3601	-1.6852	-1.1126	1	0.71538	-1.7044	1.03388
41	1	0.3931	-0.1781	-0.6219	1	-0.0697	-0.1776	0.66984
42	6	-1.1429	-0.8844	0.72877	6	1.46432	-0.748	-0.7466
43	1	-1.4569	-1.858	1.13271	1	1.79572	-1.6829	-1.2218
44	6	-0.7214	0.00563	1.90834	6	1.01221	0.21472	-1.8556
45	1	0.10889	-0.4317	2.46794	1	0.19104	-0.2019	-2.4438
46	1	-0.4004	0.99091	1.55049	1	0.66795	1.16224	-1.4249
47	1	-1.5418	0.15816	2.61362	1	1.82452	0.44033	-2.5505
48	6	-2.3435	-0.2936	-0.0378	6	2.65844	-0.1865	0.05136
49	1	-2.5251	-0.9139	-0.9247	1	2.85556	-0.8583	0.89634
50	1	-2.0697	0.70193	-0.4142	1	2.36989	0.77848	0.49099
51	6	-3.6516	-0.1918	0.7585	6	3.96015	-0.0129	-0.7429

52	1	-3.5117	0.47388	1.61482	1	3.8031	0.69747	-1.5594
53	1	-3.8984	-1.1794	1.17118	1	4.22427	-0.9703	-1.212
54	6	-4.827	0.30139	-0.0962	6	5.12961	0.45464	0.13413
55	1	-4.5959	1.30149	-0.4889	1	4.88381	1.42986	0.57717
56	1	-4.9174	-0.3546	-0.9713	1	5.23235	-0.2433	0.97467
57	6	-6.1933	0.34954	0.61751	6	6.49344	0.56016	-0.578
58	1	-6.3713	-0.6432	1.0563	1	6.68915	-0.4087	-1.0606
59	6	-7.3161	0.60976	-0.4069	6	7.61274	0.79564	0.45617
60	1	-7.1835	1.61422	-0.8326	1	7.46412	1.77916	0.92338
61	1	-7.1906	-0.0922	-1.2412	1	7.49963	0.05752	1.26039
62	6	-6.208	1.38164	1.75585	6	6.48721	1.64174	-1.6695
63	1	-6.0397	2.3906	1.36128	1	6.29805	2.62863	-1.231
64	1	-7.1636	1.38627	2.28549	1	7.44288	1.68941	-2.197
65	1	-5.4296	1.17936	2.4955	1	5.71365	1.45633	-2.4186
66	6	-8.7473	0.4712	0.12956	6	9.04558	0.70301	-0.0859
67	1	-8.8509	-0.509	0.60944	1	9.16532	-0.2553	-0.6046
68	1	-8.9318	1.21824	0.91004	1	9.21665	1.48362	-0.836
69	6	-9.8076	0.63354	-0.9679	6	10.1039	0.83943	1.01688
70	1	-9.6714	-0.151	-1.7254	1	9.98118	0.0236	1.74298
71	1	-9.6317	1.5866	-1.4831	1	9.91295	1.76852	1.56919
72	6	-11.27	0.60743	-0.4837	6	11.5661	0.8562	0.53196
73	1	-11.374	1.37285	0.29788	1	11.6568	1.65301	-0.2193
74	6	-11.664	-0.7458	0.12645	6	11.9819	-0.4656	-0.1303
75	1	-11.051	-1	0.99439	1	11.3722	-0.6961	-1.0074
76	1	-12.709	-0.7372	0.45154	1	13.0263	-0.4272	-0.455
77	1	-11.551	-1.5494	-0.6106	1	11.8832	-1.2989	0.57499
78	6	-12.223	0.97749	-1.6291	6	12.5141	1.19705	1.6908
79	1	-13.263	1.0012	-1.2881	1	13.5528	1.25109	1.35085
80	1	-11.983	1.96085	-2.0449	1	12.2582	2.1594	2.14456
81	1	-12.157	0.24587	-2.4424	1	12.4602	0.43329	2.47498
82	8	6.01419	4.70641	-0.1084	8	-8.4253	4.12422	-0.1176
83	8	5.29262	5.08978	-1.1664	8	-8.9439	4.63755	1.13873
84					1	-9.8674	4.34939	1.10302

Table S3. Calculated geometries of the complexes formed between (a) β-TOH and $O_2^{\bullet-}$, (b) β-TOH and $HO_2^{\bullet}$ in DMF, with DFT-(U)B3LYP/PCM/6-311+G(d,p).

	(a) β-TOH and $O_2^{\bullet-}$				(b) β-TOH and $HO_2^{\bullet}$			
Center Number	Atomic Number	Coordinates (Angstroms)			Atomic Number	Coordinates (Angstroms)		
		X	Y	Z		X	Y	Z
1	6	-5.314	0.456	-1.089	6	-5.37883	0.395127	-1.12477
2	6	-5.006	-0.73	-0.397	6	-4.9627	-0.76919	-0.39814
3	6	-5.42	-0.92	0.9268	6	-5.37531	-1.01134	0.93898
4	6	-6.133	0.096	1.5974	6	-6.16012	-0.07501	1.592193
5	6	-6.442	1.28	0.9097	6	-6.58324	1.12938	0.891216
6	6	-6.035	1.443	-0.421	6	-6.16532	1.306252	-0.47783
7	6	-5.092	-2.22	1.6316	6	-4.95332	-2.29624	1.614883
8	1	-4.324	-2.04	2.3949	1	-4.16792	-2.08645	2.34967
9	1	-5.969	-2.58	2.1782	1	-5.79209	-2.70285	2.184838
10	6	-4.879	0.643	-2.521	6	-4.93614	0.582523	-2.5502
11	1	-3.794	0.546	-2.626	1	-3.84557	0.609088	-2.62429
12	1	-5.325	-0.11	-3.178	1	-5.2754	-0.24477	-3.17988
13	1	-5.173	1.628	-2.887	1	-5.33571	1.513251	-2.95403
14	6	-4.631	-3.3	0.6533	6	-4.48048	-3.33861	0.599885
15	1	-4.167	-4.13	1.192	1	-3.99695	-4.17586	1.10832
16	1	-5.499	-3.71	0.1221	1	-5.3442	-3.74396	0.063456
17	6	-3.653	-2.76	-0.399	6	-3.5117	-2.75328	-0.43094
18	8	-4.314	-1.68	-1.12	8	-4.17553	-1.60491	-1.08743
19	6	-3.374	-3.82	-1.462	6	-3.25984	-3.73857	-1.56712
20	1	-4.312	-4.21	-1.86	1	-4.2067	-4.09865	-1.97494
21	1	-2.798	-3.41	-2.291	1	-2.69024	-3.27557	-2.37364
22	1	-2.807	-4.65	-1.031	1	-2.69516	-4.5962	-1.19365
23	6	-2.356	-2.23	0.253	6	-2.20977	-2.24324	0.217976
24	1	-2.621	-1.46	0.9775	1	-2.46595	-1.50542	0.984881
25	6	-1.303	-1.66	-0.706	6	-1.17293	-1.62549	-0.72959
26	1	-0.932	-2.45	-1.37	1	-0.80901	-2.38219	-1.4328
27	1	-1.776	-0.91	-1.345	1	-1.65283	-0.84574	-1.32726
28	6	-0.112	-1.05	0.0442	6	0.023904	-1.0464	0.037601
29	1	-0.464	-0.21	0.6646	1	-0.32339	-0.24035	0.698452
30	6	1.0465	-0.56	-0.846	6	1.173581	-0.51312	-0.84057
31	6	0.6151	0.603	-1.756	6	0.730427	0.682493	-1.69806
32	1	1.4274	0.928	-2.41	1	1.537785	1.038923	-2.34204
33	1	-0.225	0.324	-2.396	1	-0.11031	0.425733	-2.34692
34	1	0.3046	1.467	-1.155	1	0.417871	1.517748	-1.06047
35	6	2.262	-0.18	0.0264	6	2.392592	-0.17019	0.039509
36	1	2.0034	0.689	0.6415	1	2.13365	0.675979	0.691135
37	1	2.4477	-1.01	0.7291	1	2.586012	-1.0194	0.70702
38	6	3.5614	0.102	-0.739	6	3.686214	0.152719	-0.72055
39	1	3.7935	-0.76	-1.384	1	3.919662	-0.67779	-1.40035
40	1	3.4178	0.958	-1.404	1	3.534578	1.033637	-1.3506
41	6	4.752	0.363	0.1939	6	4.879717	0.383771	0.216273
42	1	4.538	1.241	0.8187	1	4.663409	1.235251	0.876553
43	6	6.1112	0.567	-0.506	6	6.234258	0.624809	-0.48046
44	1	6.2736	-0.29	-1.169	1	6.39901	-0.20913	-1.17841
45	6	6.1256	1.839	-1.368	6	6.236689	1.93012	-1.29125
46	1	5.3395	1.823	-2.126	1	5.446933	1.939043	-2.04625
47	1	5.9694	2.727	-0.744	1	6.078557	2.791842	-0.6321
48	6	7.2475	0.568	0.5369	6	7.375226	0.591542	0.55638
49	1	7.1326	1.447	1.1875	1	7.25665	1.441159	1.243241
50	1	7.1212	-0.31	1.1853	1	7.258351	-0.31194	1.168293
51	1	4.8433	-0.49	0.8841	1	4.979471	-0.49076	0.871588

52	1	0.2827	-1.8	0.741	1	0.425887	-1.82732	0.695333
53	1	1.3481	-1.4	-1.488	1	1.474428	-1.32506	-1.51859
54	1	7.0769	1.962	-1.891	1	7.184644	2.079875	-1.81349
55	6	8.6712	0.544	-0.036	6	8.796568	0.601989	-0.02261
56	1	8.8568	1.45	-0.624	1	8.973655	1.533868	-0.57172
57	1	8.7573	-0.3	-0.731	1	8.885347	-0.20937	-0.75443
58	6	9.745	0.437	1.0553	6	9.875553	0.454764	1.058559
59	1	9.5875	1.248	1.7786	1	9.714638	1.230236	1.818474
60	1	9.6061	-0.5	1.6133	1	9.746325	-0.50725	1.574003
61	6	11.202	0.508	0.5588	6	11.32966	0.559386	0.560509
62	1	11.307	1.43	-0.029	1	11.42614	1.509843	0.017478
63	6	12.172	0.596	1.7458	6	12.30379	0.598365	1.746768
64	1	13.208	0.686	1.4057	1	13.33789	0.712517	1.40747
65	1	11.949	1.461	2.378	1	12.07725	1.430198	2.420732
66	1	12.104	-0.3	2.3716	1	12.24522	-0.32842	2.32882
67	6	11.572	-0.67	-0.349	6	11.70449	-0.57496	-0.40463
68	1	11.457	-1.62	0.1863	1	11.59844	-1.54987	0.085257
69	1	10.946	-0.72	-1.244	1	11.07539	-0.57977	-1.29813
70	1	12.613	-0.6	-0.679	1	12.74396	-0.47959	-0.73309
71	1	-1.919	-3.05	0.8321	1	-1.763	-3.09252	0.747672
72	6	-6.564	-0.09	3.0337	6	-6.60668	-0.27712	3.012385
73	1	-6.964	0.843	3.4344	1	-7.08943	0.623021	3.387951
74	1	-7.341	-0.85	3.1295	1	-7.32324	-1.10312	3.087567
75	1	-5.729	-0.4	3.6681	1	-5.7643	-0.52627	3.663912
76	1	-6.297	2.365	-0.935	1	-6.49428	2.209748	-0.97985
77	8	-7.147	2.255	1.555	8	-7.30821	1.997034	1.467244
78	1	-7.236	3.095	0.9944	1	-8.00501	3.355634	0.754693
79	8	-7.472	4.528	0.3278	8	-8.46937	4.16583	0.409312
80	8	-7.396	4.505	-1.006	8	-7.64044	4.51646	-0.73161
81					1	-7.13986	5.270831	-0.38854

Table S4. Calculated geometries of the complexes formed between (a) γ-TOH and $O_2^{\bullet-}$, (b) γ-TOH and $HO_2^{\bullet}$ in DMF, with DFT-(U)B3LYP/PCM/6-311+G(d,p).

	(a) γ-TOH and $O_2^{\bullet-}$				(b) γ-TOH and $HO_2^{\bullet}$			
Center Number	Atomic Number	Coordinates (Angstroms)			Atomic Number	Coordinates (Angstroms)		
		X	Y	Z		X	Y	Z
1	6	-5.22019	0.261699	1.566365	6	5.356671	-0.60672	1.4813
2	6	-4.96933	0.819722	0.297819	6	4.957624	-0.93222	0.145527
3	6	-5.43483	0.198827	-0.86594	6	5.436975	-0.21455	-0.98649
4	6	-6.1502	-0.99591	-0.74871	6	6.295501	0.829769	-0.76549
5	6	-6.4135	-1.56419	0.498216	6	6.714188	1.217677	0.555771
6	6	-5.95564	-0.93121	1.670959	6	6.215043	0.452423	1.696039
7	6	-5.15664	0.802128	-2.22576	6	5.00438	-0.62795	-2.37231
8	1	-4.3923	0.20939	-2.74191	1	4.216583	0.045208	-2.72602
9	1	-6.05377	0.739373	-2.84882	1	5.840221	-0.51009	-3.06578
10	6	-4.71305	0.953955	2.810423	6	4.804174	-1.45275	2.600297
11	1	-3.96825	1.707651	2.561979	1	3.711138	-1.42309	2.606578
12	1	-5.52657	1.455625	3.348377	1	5.087876	-2.50095	2.469393
13	1	-4.26543	0.240774	3.507531	1	5.161723	-1.12083	3.572154
14	6	-6.25554	-1.53577	3.022912	6	6.670632	0.860839	3.072268
15	1	-5.35143	-1.93684	3.496423	1	5.823037	1.119339	3.713713
16	1	-6.6701	-0.79371	3.711781	1	7.217135	0.052767	3.567944
17	1	-6.96967	-2.3521	2.928645	1	7.326904	1.726321	3.00545
18	6	-4.70501	2.259664	-2.10972	6	4.513732	-2.07654	-2.37795
19	1	-4.27801	2.609538	-3.05375	1	4.048047	-2.32276	-3.33491
20	1	-5.56922	2.895932	-1.89126	1	5.365424	-2.75213	-2.25057
21	6	-3.68218	2.457578	-0.979	6	3.508401	-2.35205	-1.2536
22	8	-4.28735	2.024237	0.273675	8	4.126252	-1.97563	0.040706
23	6	-3.40115	3.9453	-0.7752	6	3.242624	-3.84751	-1.11609
24	1	-4.33851	4.49302	-0.6517	1	4.184124	-4.39524	-1.0382
25	1	-2.78781	4.11343	0.111521	1	2.643304	-4.06316	-0.23099
26	1	-2.8739	4.35313	-1.64159	1	2.703401	-4.20922	-1.99491
27	6	-2.39041	1.650697	-1.23657	6	2.216423	-1.52662	-1.41388
28	1	-2.65324	0.592306	-1.33057	1	2.479994	-0.46445	-1.43373
29	6	-1.28904	1.783702	-0.17676	6	1.133024	-1.74282	-0.34898
30	1	-0.91666	2.813813	-0.14727	1	0.760338	-2.77161	-0.39673
31	1	-1.71799	1.57731	0.807708	1	1.573532	-1.6134	0.64333
32	6	-0.1099	0.840545	-0.45323	6	-0.04724	-0.77946	-0.53667
33	1	-0.46139	-0.19999	-0.4205	1	0.304829	0.254336	-0.41695
34	6	1.095036	0.990768	0.497135	6	-1.24473	-1.01314	0.405979
35	6	0.729363	0.628019	1.944794	6	-0.86581	-0.78949	1.878208
36	1	1.575162	0.766492	2.622504	1	-1.70965	-0.97966	2.545584
37	1	-0.09067	1.244263	2.320979	1	-0.05199	-1.44783	2.191495
38	1	0.416362	-0.42061	2.010563	1	-0.53967	0.244874	2.038131
39	6	2.283602	0.15677	-0.02239	6	-2.43281	-0.12852	-0.02319
40	1	2.021768	-0.90898	0.03546	1	-2.16951	0.925316	0.143399
41	1	2.420343	0.378133	-1.08847	1	-2.57236	-0.23906	-1.10597
42	6	3.619831	0.389728	0.695757	6	-3.76759	-0.43353	0.670185
43	1	3.858904	1.461484	0.670183	1	-4.01109	-1.49503	0.527778
44	1	3.524059	0.120324	1.75137	1	-3.66729	-0.28212	1.748616
45	6	4.775613	-0.39659	0.06218	6	-4.92188	0.422404	0.131211
46	1	4.554751	-1.47186	0.114804	1	-4.69991	1.483953	0.309026
47	6	6.167451	-0.14317	0.676048	6	-6.31481	0.100727	0.709558
48	1	6.339113	0.943219	0.667094	1	-6.49144	-0.97518	0.564688
49	6	6.246636	-0.62435	2.133269	6	-6.39202	0.396101	2.215558
50	1	5.491188	-0.14395	2.759643	1	-5.64283	-0.16681	2.777383

51	1	6.087157	-1.70763	2.189099	1	-6.22187	1.462166	2.406854
52	6	7.257649	-0.79033	-0.20171	6	-7.40185	0.858032	-0.07959
53	1	7.130143	-1.88164	-0.18029	1	-7.2747	1.936949	0.086024
54	1	7.087809	-0.48546	-1.2422	1	-7.2283	0.693024	-1.15058
55	1	4.818821	-0.1477	-1.00573	1	-4.96436	0.299667	-0.95845
56	1	0.238193	1.011086	-1.48001	1	-0.40228	-0.86214	-1.57165
57	1	1.400289	2.047294	0.478157	1	-1.55534	-2.06174	0.290111
58	1	7.220757	-0.40778	2.578284	1	-7.36919	0.135438	2.629063
59	6	8.704336	-0.44158	0.173782	6	-8.84975	0.462785	0.241123
60	1	8.932755	-0.81186	1.179766	1	-9.07994	0.689708	1.288388
61	1	8.804986	0.649223	0.215809	1	-8.95172	-0.6231	0.130732
62	6	9.726692	-1.02595	-0.8102	6	-9.86951	1.180118	-0.65355
63	1	9.550738	-2.10611	-0.89403	1	-9.69622	2.261475	-0.5805
64	1	9.547515	-0.61028	-1.81167	1	-9.68511	0.913018	-1.70357
65	6	11.20502	-0.8038	-0.43793	6	-11.3487	0.903137	-0.32375
66	1	11.35261	-1.19364	0.578842	1	-11.5008	1.137989	0.738929
67	6	12.12264	-1.59663	-1.37975	6	-12.2648	1.825077	-1.14139
68	1	13.17429	-1.47928	-1.10083	1	-13.3172	1.665115	-0.88779
69	1	11.8865	-2.66495	-1.35942	1	-12.0318	2.879033	-0.96134
70	1	12.01217	-1.24991	-2.41354	1	-12.149	1.636254	-2.21475
71	6	11.59449	0.681797	-0.43418	6	-11.7348	-0.56731	-0.54151
72	1	11.4391	1.124456	-1.42494	1	-11.5754	-0.85777	-1.58646
73	1	11.00856	1.259029	0.285281	1	-11.1496	-1.24379	0.08626
74	1	12.65057	0.807161	-0.17626	1	-12.7913	-0.73155	-0.30852
75	1	-1.99839	1.956281	-2.21421	1	1.808401	-1.759	-2.40429
76	1	-6.51434	-1.49963	-1.64142	1	6.693979	1.395795	-1.60061
77	8	-7.11045	-2.73178	0.613526	8	7.504018	2.196273	0.723649
78	1	-7.46175	-3.05547	-0.281	1	8.224704	3.11666	-0.48819
79	8	-7.67684	-3.28395	-2.71594	8	8.701511	3.686244	-1.15068
80	8	-8.14733	-3.74213	-1.55203	8	7.64167	4.580257	-1.58561
81					1	7.858517	5.392582	-1.10567

Table S5. Calculated geometries of the complexes formed between (a) δ-TOH and $O_2^{\bullet-}$, (b) δ-TOH and $HO_2^{\bullet}$ in DMF, with DFT-(U)B3LYP/PCM/6-311+G(d,p).

	(a) δ-TOH and $O_2^{\bullet-}$				(b) δ-TOH and $HO_2^{\bullet}$			
Center Number	Atomic Number	Coordinates (Angstroms)			Atomic Number	Coordinates (Angstroms)		
		X	Y	Z		X	Y	Z
1	6	5.585417	0.448225	0.872997	6	5.545846	0.227373	0.977627
2	6	5.223187	-0.72842	0.190994	6	5.174271	-0.88301	0.196705
3	6	5.609103	-0.93308	-1.1385	6	5.564437	-0.98143	-1.14467
4	6	6.35258	0.058567	-1.78957	6	6.323215	0.050496	-1.70789
5	6	6.716378	1.235374	-1.13638	6	6.691909	1.158232	-0.95353
6	6	6.329389	1.417713	0.199884	6	6.303345	1.239176	0.387247
7	6	5.222752	-2.20809	-1.85513	6	5.16821	-2.18805	-1.96564
8	1	4.418803	-2.00018	-2.57074	1	4.369655	-1.91168	-2.66356
9	1	6.06728	-2.56819	-2.45013	1	6.011712	-2.5057	-2.58534
10	6	5.179701	0.646208	2.312309	6	5.134479	0.309794	2.425304
11	1	5.612752	-0.12359	2.959109	1	5.556592	-0.51641	3.005855
12	1	5.50917	1.621304	2.675412	1	5.470577	1.247379	2.871392
13	1	4.094394	0.580722	2.433481	1	4.048082	0.244349	2.533144
14	6	4.78461	-3.29066	-0.86562	6	4.715147	-3.34348	-1.06994
15	1	4.297489	-4.11831	-1.38835	1	4.22264	-4.11993	-1.66121
16	1	5.664385	-3.70217	-0.3597	1	5.588375	-3.80402	-0.59627
17	6	3.837626	-2.74005	0.213774	6	3.76761	-2.87448	0.046076
18	8	4.516498	-1.66233	0.923692	8	4.453214	-1.86116	0.844322
19	6	3.578788	-3.80078	1.282031	6	3.496515	-4.01472	1.025109
20	1	4.525035	-4.2009	1.653877	1	4.437996	-4.45069	1.367384
21	1	3.029123	-3.38282	2.126919	1	2.945407	-3.66191	1.898155
22	1	2.994811	-4.62491	0.864098	1	2.908771	-4.79796	0.539517
23	6	2.528019	-2.20335	-0.40505	6	2.465992	-2.2754	-0.52979
24	1	2.778748	-1.41608	-1.12312	1	2.726212	-1.43329	-1.17904
25	6	1.4918	-1.64755	0.580451	6	1.43154	-1.79516	0.496437
26	1	1.130795	-2.4462	1.238041	1	1.058697	-2.64399	1.080304
27	1	1.97509	-0.9067	1.223253	1	1.92011	-1.1188	1.203154
28	1	2.079834	-3.01735	-0.98774	1	2.013161	-3.03362	-1.17996
29	6	0.29008	-1.02352	-0.14269	6	0.240534	-1.09582	-0.17346
30	1	-0.11528	-1.7634	-0.84467	1	-0.16711	-1.76362	-0.94305
31	1	0.633102	-0.17873	-0.75586	1	0.595451	-0.20138	-0.70397
32	6	-0.85608	-0.54478	0.770944	6	-0.90709	-0.69284	0.774281
33	1	-1.14799	-1.39396	1.40614	1	-1.21809	-1.59707	1.317703
34	6	-0.41248	0.604582	1.689166	6	-0.45504	0.350485	1.807823
35	1	0.437261	0.318031	2.313311	1	0.37878	-0.014	2.412539
36	1	-0.11209	1.476172	1.095887	1	-0.12845	1.269844	1.307768
37	1	-1.21534	0.918897	2.360298	1	-1.262	0.613985	2.495683
38	6	-2.08379	-0.16124	-0.07984	6	-2.1202	-0.20422	-0.04292
39	1	-2.28036	-0.97615	-0.78803	1	-2.31466	-0.9316	-0.84125
40	1	-1.83293	0.717735	-0.68991	1	-1.85408	0.735702	-0.54637
41	6	-3.37203	0.118452	0.706146	6	-3.41615	-0.00334	0.754395
42	1	-3.21976	0.970829	1.374234	1	-3.26136	0.754932	1.527124
43	1	-3.59397	-0.74358	1.349675	1	-3.66136	-0.93587	1.280408
44	6	-4.57595	0.383587	-0.20808	6	-4.60112	0.397011	-0.13506
45	1	-4.37064	1.264163	-0.83274	1	-4.37674	1.352416	-0.62968
46	1	-4.67746	-0.46215	-0.89992	1	-4.69776	-0.34494	-0.9378
47	6	-5.92483	0.586311	0.511458	6	-5.96122	0.51437	0.582273
48	1	-6.07646	-0.27619	1.176952	1	-6.13929	-0.43502	1.1083
49	6	-7.0764	0.586194	-0.51416	6	-7.09093	0.688321	-0.45273
50	1	-6.97085	1.463343	-1.16773	1	-6.96142	1.653713	-0.96156
51	1	-6.95976	-0.29128	-1.16286	1	-6.97101	-0.08081	-1.22634

52	6	-5.92794	1.857991	1.374003	6	-5.96345	1.642663	1.625466
53	1	-5.78369	2.746615	0.748257	1	-5.78865	2.611739	1.143387
54	1	-6.87102	1.979216	1.91227	1	-6.9173	1.70152	2.155147
55	1	-5.1297	1.842987	2.119948	1	-5.18429	1.49969	2.377977
56	6	-8.49166	0.563134	0.079221	6	-8.51857	0.595293	0.102867
57	1	-8.56712	-0.27741	0.778907	1	-8.61938	-0.3426	0.661335
58	1	-8.66946	1.471059	0.666903	1	-8.6976	1.403387	0.821344
59	6	-9.58078	0.451203	-0.99601	6	-9.58594	0.66896	-0.99723
60	1	-9.44954	-0.48798	-1.5515	1	-9.45354	-0.17341	-1.69056
61	1	-9.43374	1.258195	-1.7253	1	-9.41451	1.578041	-1.58791
62	6	-11.0304	0.52364	-0.47944	6	-11.0452	0.680304	-0.50332
63	1	-11.1282	1.449718	0.103968	1	-11.1449	1.504813	0.216266
64	6	-11.3869	-0.65309	0.440809	6	-11.434	-0.6209	0.213912
65	1	-10.7481	-0.69103	1.326563	1	-10.8149	-0.80608	1.09511
66	1	-12.4234	-0.57931	0.784061	1	-12.4768	-0.58725	0.544205
67	1	-11.2795	-1.60564	-0.09097	1	-11.3255	-1.47986	-0.45849
68	6	-12.017	0.604836	-1.65321	6	-12.0061	0.958388	-1.66821
69	1	-13.0484	0.696312	-1.29908	1	-13.0435	1.008289	-1.32349
70	1	-11.8037	1.466135	-2.29365	1	-11.7697	1.90607	-2.16168
71	1	-11.9575	-0.29606	-2.27449	1	-11.9439	0.164955	-2.42171
72	1	6.620011	2.332721	0.710095	1	6.592876	2.100297	0.981354
73	1	6.659338	-0.09083	-2.82034	1	6.636367	-0.0138	-2.74492
74	8	7.439754	2.166238	-1.81674	8	7.435763	2.137935	-1.56578
75	1	7.661209	2.977293	-1.24547	1	7.661409	2.840931	-0.93276
76	8	8.129088	4.330434	-0.55782	8	7.721971	5.44325	-0.28311
77	8	7.754442	4.435801	0.720565	1	8.064547	6.156171	0.294619
78					8	8.250556	4.318577	0.168432
